# Tuberculosis Treatment Outcome and Predictors in Africa: A Systematic Review and Meta-Analysis

**DOI:** 10.3390/ijerph182010678

**Published:** 2021-10-12

**Authors:** Melese Yeshambaw Teferi, Ziad El-Khatib, Minyahil Tadesse Boltena, Azeb Tarekegn Andualem, Benedict Oppong Asamoah, Mulatu Biru, Hawult Taye Adane

**Affiliations:** 1 Armauer Hansen Research Institute, Ministry of Health, Addis Ababa P.O. Box 1005, Ethiopia; minyahil.tadesse@ahri.gov.et (M.T.B.); azititar@gmail.com (A.T.A.); mulatu.biru@ahri.gov.et (M.B.); hawultachew@gmail.com (H.T.A.); 2 Department of Global Public Health, Karolinska Institutet, 171 77 Stockholm, Sweden; ziad.khatib@gmail.com; 3 Department of Clinical Sciences, Social Medicine and Global Health, Lund University, 221 00 Lund, Sweden; benedict_oppong.asamoah@med.lu.se

**Keywords:** tuberculosis, treatment outcomes, resource-limited settings, systematic review, meta-analysis

## Abstract

This review aimed to summarize and estimate the TB treatment success rate and factors associated with unsuccessful TB treatment outcomes in Africa. Potentially eligible primary studies were retrieved from PubMed and Google Scholar. The risk of bias and quality of studies was assessed using The Joanna Briggs Institute’s (JBI) appraisal criteria, while heterogeneity across studies was assessed using Cochran’s Q test and I2 statistic. Publication bias was checked using the funnel plot and egger’s test. The protocol was registered in PROSPERO, numbered CRD42019136986. A total of 26 eligible studies were considered. The overall pooled estimate of TB treatment success rate was found to be 79.0% (95% CI: 76–82%), ranging from 53% (95% CI: 47–58%) in Nigeria to 92% (95% CI: 90–93%) in Ethiopia. The majority of unsuccessful outcomes were attributed to 48% (95% CI: 40–57%) death and 47% (95% CI: 39–55%) of defaulter rate. HIV co-infection and retreatment were significantly associated with an increased risk of unsuccessful treatment outcomes compared to HIV negative and newly diagnosed TB patients with RR of 1.53 (95% CI: 1.36–1.71) and 1.48 (95% CI: 1.14–1.94), respectively. TB treatment success rate was 79% below the WHO defined threshold of 85% with significant variation across countries. Countries need to explore contextual underlining factors and more effort is required in providing TB preventive treatment, improve case screening and linkage for TB treatment among HIV high-risk groups and use confirmatory TB diagnostic modality. Countries in Africa need to strengthen counseling and follow-up, socio-economic support for patients at high risk of loss to follow-up and poor treatment success is also crucial for successful TB control programs.

## 1. Introduction

Tuberculosis (TB) is a global public health problem that typically affects the lungs while extrapulmonary TB (EPTB) infects other organs of the body [1]. TB is one of the top ten causes of death in the world [2]. TB-related deaths by 2019 were estimated to be 1.2 million among HIV-negative and 208,000 among HIV-infected persons. Globally, 10 million new cases of TB have been recorded in 2019 [3], however pediatric TB appears to be increasing, particularly in low- and middle-income countries [4]. Almost 90% of primary global TB cases each year occur in 30 high TB burden countries in Africa and about 87% of TB patients in this region were HIV co-infected which requires an evidence-based public health intervention [2,5].

The World Health Organization (WHO) report of 2020, shows that the global treatment success rate of new cases of TB was 85% and 76% for TB patients living with HIV [3]. However, the TB success rate was found to be 78.9% in Africa, and 80.1% globally [6]. An epidemiological study conducted on pediatric TB in SSA reported a treatment success rate of 69.6% [7]. Africa has the highest regional TB prevalence with high TB/HIV comorbidity, a significant factor in the TB epidemic and associated mortality [1]. Reports from Africa identified several factors associated with poor TB treatment success rate such as including old age group [8,9,10,11], HIV infection [8,10,12,13,14,15,16,17] sputum smear positivity [11,15,17], and previous TB treatment [8,12,18]. A study conducted in Africa outcomes also reported that HIV status is a predictive factor for TB-related mortality [19].

The TB treatment coverage is an indicator for achieving the goals of the End TB Strategy [20]. The treatment success of 85% reported by WHO 2020, shows that 90% of the End TB strategy will be unattainable without evidence-based planning and policy development [20,21]. Rigorous evidence to understand the gap in the current TB treatment success and associated risk factors in Africa is needed to guide the TB prevention and control program. Therefore, this systematic review and meta-analysis aimed to produce pooled estimates of TB treatment success rate and determine the main contextual factors associated with poor TB treatment outcomes in Africa.

## 2. Methods

### 2.1. The Review Approach and Protocol Development

The review protocol has been registered in the International Prospective Register of Systematic Reviews (PROSPERO) with a CRD42019136986 registration number. The (Condition Context Population (CoCoPo)) review method was used to design the overall review approach. The Preferred Reporting Items for Systematic Reviews and Meta-Analyses (PRISMA) guideline was used to review and report each section of the article [22].

### 2.2. Search Strategies

The literature search was conducted from August to September 2020 and articles were retrieved up to 15 September 2020. Articles published in the English language and conducted in Africa since 2010 were considered eligible for this review. Original studies providing information on our outcome interest were identified through a computerized systematic search using PubMed, Google Scholar, and Science Direct databases. Terms within the same concepts were connected with Boolean operators “OR” and combined with other search terms using Boolean operators “AND”. Through that, the final search term was built in the PubMed database, which was the primary search engine used (File S1). A combination of keywords and search terms were also used to identify studies from other databases such as Google Scholar. References of identified studies were searched to avoid the exclusion of relevant articles.

### 2.3. Study Selection Process

After the removal of duplicates using Endnote, three levels of screening based on title, abstract, and full-text review were performed. Articles that were not fulfilling the criteria were excluded at any level of the title, abstract or full-text review. A full-text review was conducted for articles that pass the title and abstract review. A detailed full-text review was conducted to find out potential articles with TB treatment outcomes and factors affecting TB treatment success. Studies with our intended outcome of interest were identified as eligible. The methodological quality assessments were conducted using the JBI quality appraisal checklist, and studies judged to be of high quality were included in the analysis.

### 2.4. Inclusion and Exclusion Criteria

• Inclusion criteria

Observational studies which reported TB treatment outcomes and were conducted in Africa that are published in the English language within the last 10 years were included in the review. Outcomes were reported according to the WHO definition of treatment success (cure or treatment completion), failure, default, and death.

• Exclusion Criteria

Studies that focused on treatment outcome of patients with MDR-TB and both MDR-TB cases and drug-susceptible TB cases together and studies for which full articles not accessible were excluded. Studies conducted outside Africa, systematic reviews, randomized controlled trials, and experimental studies were excluded.

### 2.5. The Operational Definition of TB Treatment Outcomes

According to the WHO standard definition and the national guideline in most high-burden countries, TB treatment success rate was defined as the sum of cured and completed treatment. Cured refers to those patients with bacteriological confirmed TB at the beginning of treatment with smear- or culture-negative in the last month of treatment and on at least one previous occasion. Yet, patients who completed treatment without evidence of failure but with no record to show that sputum or culture results in the last month of treatment and on at least one previous occasion were negative, either because tests were not done or because results were unavailable, therefore they were considered as treatment completed. All other unfavorable outcome measures that included treatment failure, died, loss to follow up and those moved to Multi-Drug Resistance (MDR) and patients for whom no treatment outcome is assigned such as “transferred out” cases with the unknown outcomes at reporting unit were known to be unsuccessful treatment outcome [23].

#### Outcome Definition

- Cured: A pulmonary TB patient with bacteriologically confirmed TB at the beginning of treatment who was smear- or culture-negative in the last month of treatment and on at least one previous occasion.
- Treatment completed: A TB patient who completed treatment without evidence of failure BUT with no record to show that sputum smear or culture results in the last month of treatment and on at least one previous occasion were negative, either because tests were not done or because results are unavailable.
- Treatment failed: A TB patient whose sputum smear or culture is positive at month 5 or later during treatment.
- Died: A TB patient who dies for any reason before starting or during treatment.
- Lost to follow-up: A TB patient was not initiated on TB treatment or whose treatment was interrupted for two consecutive months or more.
- Treatment success: The sum of cured and treatment completed.
- Unsuccessful Treatment outcome: A sum of treatment failure, died and defaulter.

### 2.6. Data Extraction and Review Process

All of the articles that were identified from searches of the electronic databases were imported into the EndNote and duplicates were removed. Before the data extraction, full-length articles of the selected studies were read to confirm the fulfillment of the inclusion criteria. Data were extracted by two authors (MYT and HTA) independently. The data such as year of publication, author(s), the geographical location of the study area, the period of study, study design, sample size, HIV status, TB treatment outcomes, and factors affecting TB treatment outcome were extracted. Disagreements between reviewers in data extraction were resolved by the third person (MTB) to reach the final decision.

### 2.7. Methodological Quality Assessment

The two authors (MYT and HTA) independently assessed the methodological quality of included studies. The risk of bias and the overall quality of included studies was evaluated according to the Joanna Briggs Institute (JBI) quality appraisal tool [24]. The JBI critical appraisal checklist for cohort and cross-sectional studies were used. Studies scoring 6 and above out of the 9 criteria were included in the systematic review section and statistical estimation of overall treatment success rate. Studies with clear and complete numerical data were considered for further meta-analysis reports that were used to identify the contribution of individual study variables attributed with unsuccessful treatment outcomes (Table S1).

### 2.8. Statistical Analysis

Meta-analysis was carried out using metaprop command of STATA version 14 (StataCorp LP, College Station, TX, USA) that used to estimate the pooled and individual study treatment success rate. That was also used to estimate the proportion of unsuccessful treatment outcomes attributed to death, failure, and defaulter. Using metan command we had measured the relative contribution of factors (age, sex, HIV co-infection, TB retreatment status, and type of TB expected to have an association with unsuccessful TB treatment outcomes. All of the meta-analysis estimates (ES), the proportion and RR, with 95% confidence intervals (CI), and the respective weight of individual studies had been reported. Heterogeneity between studies was assessed using Cochran’s Q test and the *I*^2^ statistic. A detailed description of the original studies was presented (Table S2).

## 3. Results

### 3.1. Search Results

**Figure 1:**
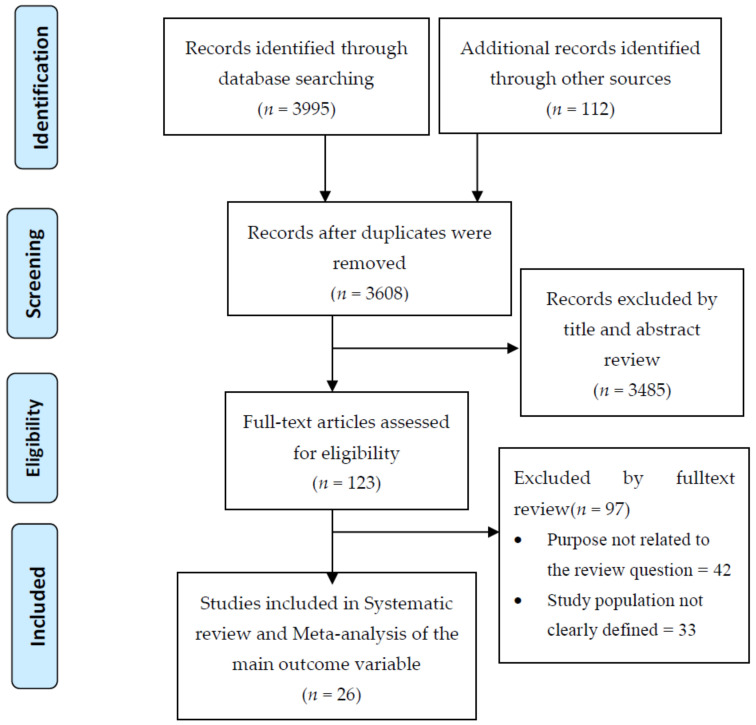
PRISMA flow diagram shows the searching strategy and screening of eligible studies.

This review included studies conducted in SSA which were published from 2010 to 2020. A total of 4107 citations were identified and exported to EndNote (Figure 1). After duplicates were removed 3608 articles were remained and then subjected to further screening and eligibility assessment. Of which 3485 of the studies were excluded based on title and abstract review, thus, based on the two independent reviewer’s agreements, 123 were to be retained on detailed full-text review. After full-text evaluation, 26 articles were found to be eligible and were included in this review (Figure 1).

### 3.2. Characteristics of Study Setting and Context

Over half of the articles (61.5%) used cohort study design [7,9,13,14,25,26,27,28,29,30,31,32,33,34,35,36] whereas the rest 38.8% were retrospective cross-sectional studies [10,37,38,39,40,41,42,43,44] (Table 1). A total of 306,351 study participants with a sample size ranging from 227 in Ethiopia [41] to 170,017 in South Africa [37]; for studies conducted in Ethiopia and South Africa respectively. Most of the study populations were from regions that are currently listed under the WHO as high TB burden countries [7,9,10,13,14,26,27,28,29,30,32,33,36,37,38,39,40,41,42,43,44,45].

### 3.3. Characteristics of the Study Population

From a total of 306,351 included study participants, 182,745 (59.7%) of them were adults while children accounted for the remaining 123,606 (40.3%). The majority 163,126 (53.2%) of patients were male. Because of the two largest studies that included settings with a high prevalence of TB/HIV co-infection [25,31], the proportion of TB-HIV co-infection participants were extremely higher 165,551 (54.0%). The percentage of retreatment cases compared to newly diagnosed TB patients was found to be reasonable, with about 16,492 (5.4%) of the total participants included in the current study. However, the clinical and demographic-based patient classification varies from study to study and some of the studies did not report findings of those basic study characteristics (Table S2).

### 3.4. Characteristics of the Condition

Out of the total 319,224 participants included from the original studies, 260,407 (85.0%) of them had a successful TB treatment outcome; which is the sum of cured and completed cases. The TB treatment success rate varied from the lowest 157/299 (52.5%) reported in Nigeria [28], to the highest prevalence rate 914/995 (91.9%) reported from Ethiopia [39]. In some of the studies, some participants were on treatment and it is known that the treatment outcome for those who were transferred out did not yet confirm. Because we had summarized the proportion of unsuccessful treatment outcomes here refers to the defaulter, treatment failure, and death; which represents 44.6%, 4.6%and 50.9% respectively. However, the detailed treatment outcome status; including extracted data for transferred out and characteristics of individual studies had been summarized and documented as a supplementary file (Table S2).

### 3.5. Factors Associated with TB Treatment Outcome

Studies reported factors that were strongly associated with a higher risk of poor TB treatment success. HIV co-infection [9,13,27,29,30,32,33,34,36,41], residence in rural areas [10,14,39,43], and previous TB treatment [32,43] were frequently reported and found to be strongly associated with high unsuccessful TB treatment outcome. Some of the studies observed poor treatment success rate among smear-negative PTB-patients [10,31,32] and extrapulmonary TB (EPTB) patients [9,10,31,32] as compared to the smear-positive PTB-patients. It is expected that HIV co-infection had been reported as an independent predictive factor for the deaths of TB patients while they were on treatment [14,28,35,44].

### 3.6. Meta-Analysis

#### 3.6.1. The Overall TB Treatment Success Rate

**Figure 2:**
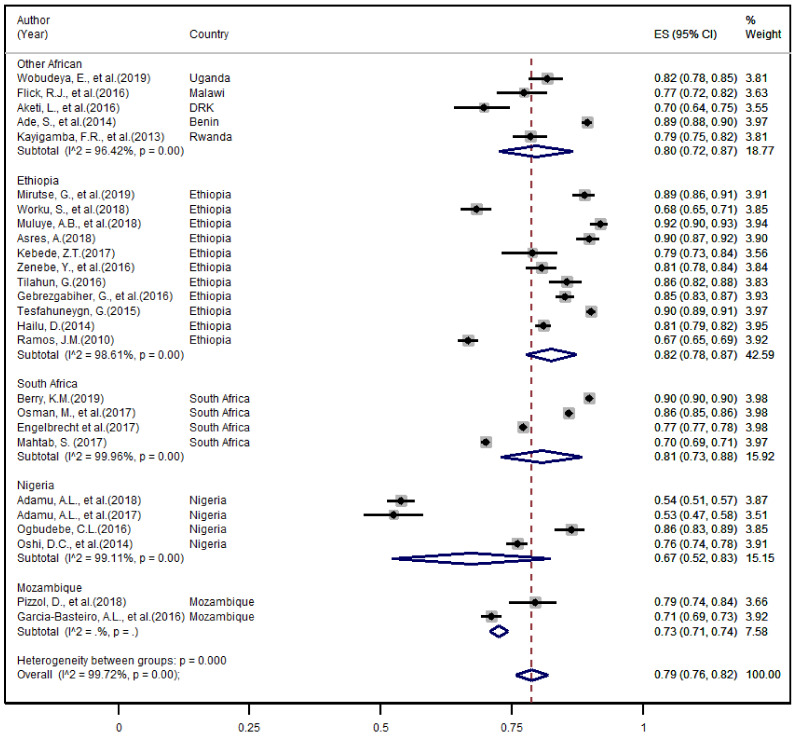
The overall pooled and region-based subgroup estimates of treatment success rate.

The overall treatment success rate for the drug-sensitive TB was 79.0% (95% CI: 76–82%) (Figure 2). The heterogeneity test indicated that all studies on successful treatment outcomes had significant heterogeneity (*I*^2^ = 99.72, *p* < 0.001), and therefore the random-effect model was used for the meta-analysis. The subgroup analysis indicated Nigeria has the lowest treatment success rate at 67% (95% CI: 52–83%), followed by Mozambique 73% (95% CI: 71–74%), Other African Countries 80% (95% CI: 72–87%), South Africa 81% (95% CI: 73–88%), and Ethiopia 82% (95% CI: 78–87%) (Figure 2).

As described above, we had estimated the overall pooled and study-specific rate of unsuccessful treatment outcomes that attributed with death, defaulter, and treatment failure. Accordingly, the corresponding pooled estimate (ES) found to be 48% (95% CI: 40–57%), 47% (95% CI: 39–55%) and 6% (95% CI: 4–7%), respectively (Figures S1–S3). Indicated that the majority of poor outcomes were due to death and defaulter or non-adherence while only a few; nearly 6% of the cases were because of inadequate treatment or failure. As compared to other African countries, subgroup analysis reflected that the contribution of death was relatively higher in Ethiopia 53% (95% CI; 35–72%) while a relatively lower rate of treatment failure 3% (95% CI: 1–4%) has been observed in this country (Figures S1 and S3). On the other hand, a relatively higher rate of defaulters or poor treatment adherence was documented among patients treated in South Africa 47% (95% CI: 39–55%) (Figure S2).

#### 3.6.2. Factors Associated with Unsuccessful TB Treatment Outcome

**Figure 3:**
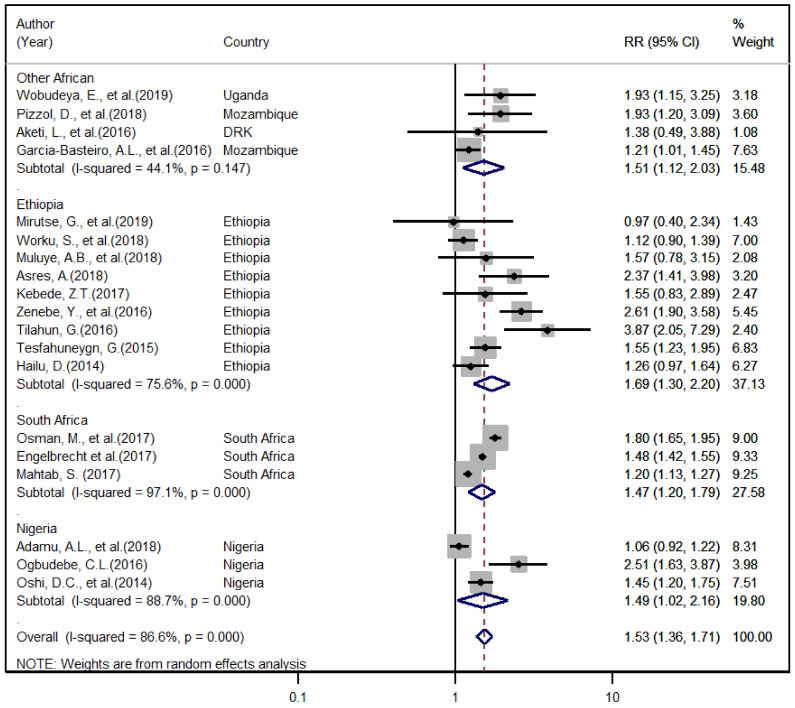
The overall and region-based relative risk of unsuccessful treatment outcome that can be associated with HIV infection.

**Figure 4:**
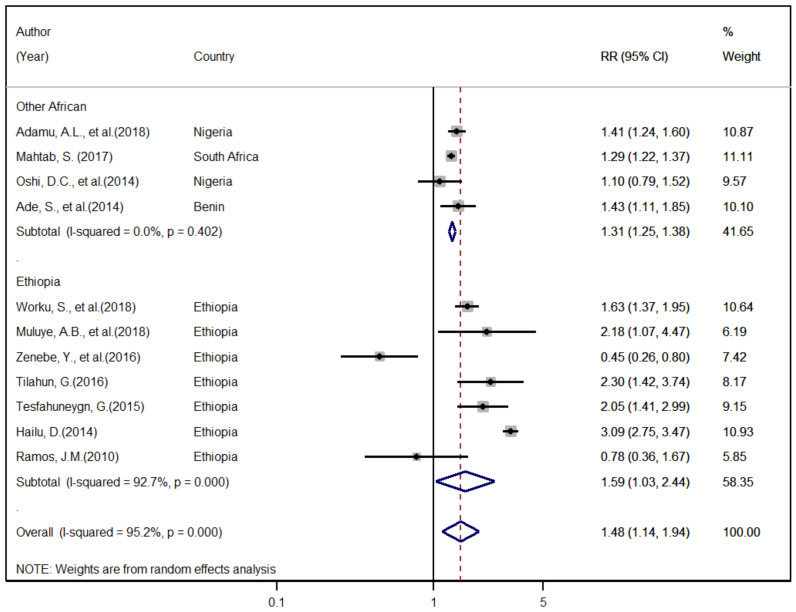
The overall and region-based relative risk of unsuccessful TB treatment outcome that can be associated with retreatment.

The demographic and clinical factors expected to have a relationship with the TB treatment success rate were assessed. The association of HIV co-infection and retreatment status were assessed using 19 articles [7,9,13,14,25,26,27,29,30,32,33,38,39,40,41,42,43,44,45] reported tractable data on HIV co-infection and 11 articles [9,14,29,32,33,34,36,38,39,40,41] with data on retreatment used for analysis. Accordingly, the overall pooled and region-based subgroup estimates indicated that HIV co-infection and retreatment were significantly associated with an increased risk of unsuccessful treatment outcome; as compared to HIV negative and newly diagnosed TB patients with RR of 95% CI: (1.53 (1.36, 1.71)) and (1.48 (1.14, 1.94)), respectively (Figure 3 and Figure 4). However, the results of this analysis showed that there was no observed significant association of TB treatment success with age, sex, and TB type.

#### 3.6.3. Publication Bias

Publication bias was assessed using the funnel plot (Figure S4) constructed from study estimates with a pseudo 95% confidence limit against the standard error of the estimates. According to this plot, the reviewed studies were symmetrically distributed representing the pooled estimate, suggesting minimal publication bias. Similarly, egger’s test indicated that there is no significant publication bias on studies reporting TB treatment success in Africa (*p* = 0.016).

## 4. Discussion

The review was conducted to estimate the pooled TB treatment success rate and predictive factors of patients with drug-susceptible TB in Africa. Data from 26 studies were analyzed to determine the pooled treatment success rate of drug-susceptible patients. The current review found that the pooled treatment success rate for drug-sensitive TB was 79.0% (95% CI: 76–82%). This pooled estimate falls below the 85% treatment success rate of the WHO 2020 report and lagging behind a 90% reduction in the TB incidence rate by 2035 [3]. The finding of our review is consistent with a finding of global review that produced 80.1% pooled estimates of TB treatment outcome in adults [6]. The current result was slightly higher compared with the 76.2% pooled treatment success rate of TB in SSA [46].

The low treatment success rate of this study compared with WHO 2020 report may be attributed to the fact that nearly all of the studies included in this meta-analysis were among the three high TB-burden countries countries [7,9,10,13,14,26,27,28,29,30,32,33,36,37,38,39,40,41,42,43,44,45] and countries with high TB/HIV co-infection [25,31]. More than one-fives of the analyzed studies were also conducted before and during 2015 [9,32,33,34,35,36]. Most of the included studies in the review were from low socio-economic settings, where poor income status directly or indirectly affects the treatment success rate of TB patients [26,47,48]. Previous studies suggested that without adequate resources that assure equitable access to diagnosis and care to address determinants of TB, countries could not reach and cure everyone [49,50]. Thus, strengthening the health care system, integrating TB services into existing programs evidence-based policies, and contextualized strategy, are fundamental to improving TB prevention and control services [51,52].

A further possible explanation for this low TB treatment success rate may be attributed to diagnosis delay, treatment delay, low detection rate, and presence of untreated infectious TB cases in the community [13,53]. Health care utilization of the community and accessibility of health care services may also possible reasons for low treatment success rates [54,55,56]. The finding is an indication that health promotion, improving diagnostic capacity, early treatment, and prevention activities are fundamental to reduce TB incidence and mortality [13,57]. TB preventive treatment (household contacts and people living with HIV), prevention of transmission of M. tuberculosis, vaccination of children and increase bacteriologically confirmed cases need to improve the TB treatment outcome [3]. Maintaining close monitoring and provision of socio-economic support to patients at high risk of poor treatment success is also crucial for successful TB control programs [11,48].

The current review found a considerable difference in TB treatment success rate across countries in subgroup analyses from 67% (95% CI: 52–83%) in Nigeria to 82% (95% CI: 78–87%) in Ethiopia. This variation across countries could be as a result of the difference in participants involved in the included study, the study area, prevalence of HIV co-infection, the extent of health care utilization within the community, as well as accessibility of health care facilities. The higher TB treatment success rate of Ethiopia (82%, 95% CI: 78–87%) compared with others in the subgroup analyses was similar to the outcome of a review conducted in Ethiopia by 2018, 83.7% [12]. However, the finding of the current review reported a low TB success rate in Ethiopia compared with the WHO 2020 TB report of 88% success rate of the country’s [3]. This may show the substantial difference between the research and programmatic reports. The review also showed a higher pooled estimate compared with a review conducted in Ethiopia among children with a success rate of 79.62% [58]. The observed differences of reports may show those study participants, time, and type of reports have a substantial contribution to the TB treatment success rate.

The low treatment success rate of Nigeria in the subgroup analyses may be due to differences in the study participants and HIV prevalence within the study areas. The finding of this review shows a 67% (95% CI: 52–83%) low TB treatment success rate in Nigeria compared with 87% WHO 2020 TB report of the country [3]. Similarly, the pooled estimate of the TB treatment success rate of Nigeria was low compared with the 83.7% reported by a review conducted in Ethiopia [59]. This difference in TB treatment success rate may be due to the time spent on the health care utilization and geographical difference. The other potential reason may also be that the TB treatment success in Nigeria was affected by a high proportion of HIV co-infection in which almost one-third of the participants were HIV co-infected in two included studies [14,28]. Nigeria was one of the 30 high TB burden countries with the lowest levels of treatment coverage in 2019 (50% or less) and accounted for 11% of the global gaps of underreporting of TB cases which may also be the potential reason for this low TB treatment outcome [3]. The type of reports may also be a reason for the difference in TB treatment success rate as there is observed variability between programmatic and research reports. This significant difference in TB treatment success rate across countries is an indicator that TB treatment needs contextual strategy and evidence-based prevention control strategy.

The rate of unsuccessful treatment outcomes that attributed with death, defaulter, and treatment failure was found to be 48% (CI: 40–57%), 47% (CI: 39–55%), and 6% (4–7%), respectively. This indicated that the majority of poor outcomes were due to death and defaulter or non-adherence whiles only a few; nearly 6% of the cases were because of inadequate treatment or failure. The current finding is supported by the recent review conducted in SSA which reported a high contribution of lost to follow-up and dearth, 42%, and 32% respectively [46]. Similarly, the reviews in SSA and Ethiopia reported a high defaulting rate within a range of 11.3–29.6% [60,61]. However, an article from a resource-limited setting identified 9.4% and 9.9% TB treatment default rates and death rates respectively [62]. This high contribution of defaulter rate and death in unsuccessful treatment outcomes may be an indicator of poor DOT implementation and patient counseling in the study area. This finding suggests that evidence-based integrated interventions, such as patient education and counseling, incentives and enablers, establish reminders and tracer platforms, and digital health technologies need to be applied to improve TB treatment success [56,63].

As compared to other African countries, subgroup analysis reflected that the contribution of death was relatively higher in Ethiopia 53% (95% CI: 35–72%) while a relatively lower rate of treatment failure 3% (95% CI: 1–4%) has been observed in this country. The finding of this review identified an 82% high overall TB treatment success rate in Ethiopia compared to other African countries. However, this country was found with a relatively higher death rate contributing to unsuccessful treatment outcomes which may need further assessment. On the other hand, a relatively higher rate of defaulters or poor treatment adherence was documented among patients treated in South Africa 56% (95% CI: 35–77%). This high defaulting rate is in line with the reports from SSA and South Africa with 42% and 29% high defaulting rates [46,61]. The high defaulting rate may be due to patient-level barriers including limited knowledge, attitudes, and beliefs regarding TB, and economic burdens and system-level barriers including centralization of services, health system delays, and geographical access to healthcare [61].

The current study indicated that HIV co-infection was a predictive factor for poor TB treatment success with (OR = 1.53; 95% CI: 1.36–2.72; *p* = 0.001) HIV co-infected patients were twice more likely to have poor treatment success compared with their HIV negative counterparts. These results agree with the findings of other studies, which reported that HIV co-infection significantly affects the overall TB treatment success rate [6,12,58,59,64,65,66]. The finding of our review also supported studies that reported being HIV positive as a predictive factor for TB-related mortality [8,14,28,35,44]. The clinical history of previous TB treatment was also identified as a factor of poor TB treatment success comparable with HIV co-infection. Retreatment cases were one and half times more likely to have poor TB treatment success compared with new TB cases (OR = 1.48; 95% CI: 1.14–1.94; *p* = 0.001). This finding is consistent with the reports from different countries [6,59,67,68].

### Limitations of the Study

Despite such imperative findings, our review had some limitations. The findings are based exclusively on observational studies with some methodological differences in the study designs which could potentially compromise the findings. The treatment outcome of transferred outpatients was not determined since most of the included studies had not defined the outcome of transferred out cases. The high degree of heterogeneity among the studies was also another limitation of the review.

## 5. Conclusions

The main goal of the current study was to produce pooled estimates of TB treatment outcomes and determine associated factors in low- and middle-income countries. Participants’ data from 26 systematically searched studies were used for the analysis. The finding of this review has shown that the TB treatment success rate in resource-limited settings was 79% below the WHO-defined threshold of 85%. Significant variations were observed in TB treatment success rates across countries. The finding identified a high contribution of loss to follow-up and death for unsuccessful TB treatment. HIV co-infection and history of previous TB treatment were predictors of poor TB treatment success. The evidence from this study suggests that countries in resource-limited settings warranted attention to improving TB treatment success rate particularly; there is a need to explore contextual underline factors. Providing TB preventive treatment (household contacts and people living with HIV), improve case screening and linkage for TB treatment among HIV high-risk groups, and confirming diagnostic modality is fundamental to improve TB treatment outcomes. Strong counseling and follow-up, socio-economic support for patients at high risk of loss to follow-up, and poor treatment success are also crucial for successful TB control programs.

## Figures and Tables

**Table 1:** Characteristics of included studies for systematic review and meta-analysis.

ID	Author (Year)	Country	Sample Size	Overall Success		Treatment Success Rate Among Different Groups									
				No	%	Child	Adult	Male	Female	HIV Pos	HIV Neg	Retreatm	New TB	EPTB	PTB
1	Wobudeya, E. et al., (2019) [25]	Uganda	516	422	81.78	81.78		83.80	79.31	61.29	83.09				
2	Mirutse, G. et al., (2019) [45]	Ethiopia	840	746	88.81	88.81		92.14	87.14	91.23	90.94			91.43	84.18
3	Berry, K.M. (2019) [37]	South Africa	17007	15262	89.80		88.97								
4	Worku, S. et al., (2018) [38]	Ethiopia	985	672	68.22	61.62	68.96	66.67	69.94	65.13	69.97	29.41	66.11	69.97	67.20
5	Muluye, A.B. et al., (2018) [39]	Ethiopia	995	914	91.86	90.91	91.99	90.27	94.13	88.24	92.80	81.58	92.33	92.54	91.34
6	Asres, A. (2018) [13]	Ethiopia	699	627	89.70		89.70	89.67	89.74	75.81	91.05		89.70	89.93	89.25
7	Adamu, A.L. et al., (2018) [14]	Nigeria	1381	745	53.95		53.95	92.28	24.84	52.00	55.89	33.82	60.52	56.82	
8	Pizzol, D. et al., (2018) [26]	Mozambique	301	239	79.40					70.23	86.47				
9	Osman, M. et al., (2017) [27]	South Africa	29,519	25,353	85.89	85.89				78.08	88.88				
10	Engelbrecht et al., (2017) [42]	South Africa	66,940	51,668	77.19	77.19		74.38	80.04	75.52	84.70		77.19		
11	Mahtab, S. (2017) [40]	South Africa	12,672	8870	70.00			69.53	70.55	66.56	73.50	62.50	73.28	67.20	71.78
12	Kebede, Z.T. (2017) [41]	Ethiopia	227	179	78.85	78.85				75.00	85.19				
13	Adamu, A.L. et al., (2017) [28]	Nigeria	299	157	52.51	52.51									
14	Flick, R.J. et al., (2016) [31]	Malawi	295	228	77.29	77.29									
15	Aketi, L. et al., (2016) [7]	DRK	283	197	69.61	69.61				57.14	72.31			69.81	70.49
16	Zenebe, Y. et al., (2016) [43]	Ethiopia	671	542	80.77	87.50	80.26	84.02	75.58	61.33	88.03	91.18	78.13	76.83	84.55
17	Tilahun, G. (2016) [29]	Ethiopia	491	420	85.54	85.54		85.84	85.29	70.73	93.78	59.46	85.68	86.83	84.27
18	Garcia-Basteiro, A.L. et al., (2016) [44]	Mozambique	1957	1393	71.18					71.03	77.22				
19	Ogbudebe, C.L. (2016) [30]	Nigeria	555	479	86.31	95.24	83.90	86.71	81.17	71.28	90.24				
20	Gebrezgabiher, G. et al., (2016) [10]	Ethiopia	1537	1310	85.23	83.17	83.75	85.99	84.03					81.92	85.66
21	Tesfahuneygn, G. (2015) [32]	Ethiopia	4275	3853	90.13		90.13	89.73	90.70	84.50	90.54	78.18	90.44	92.08	87.54
22	Hailu, D. (2014) [33]	Ethiopia	2708	2193	80.98	80.98		81.81	81.15	80.06	84.76	4.97	81.27	81.96	80.73
23	Oshi, D.C. et al., (2014) [9]	Nigeria	1668	1268	76.02		76.02	74.45	78.16	65.79	78.66	73.28	76.22	45.74	
24	Ade, S. et al., (2014) [34]	Benin	3714	3319	89.36							84.87	89.89	86.42	
25	Kayigamba, F.R. et al., (2013) [35]	Rwanda	581	457	78.66		78.66								
26	Ramos, J.M. (2010) [36]	Ethiopia	2225	1484	66.70	66.38	66.97	81.48	84.45			87.50	83.35	83.38	83.23

## Data Availability

The datasets during and/or analyzed during the current study are available from the corresponding author on reasonable request.

## Supplementary Materials

The following are available online at https://www.mdpi.com/article/10.3390/ijerph182010678/s1, File S1: Systematic Electronic search, Table S1: PRISMA 2009 checklist. Study level risk of bias measurement, Table S2: Characteristics of included studies and TB treatment outcome status, Figure S1: Pooled and study-specific rate of unsuccessful treatment outcome related to death, Figure S2: Pooled and study-specific rate of unsuccessful treatment outcome related to the defaulter, Figure S3: Pooled and study-specific rate of unsuccessful treatment outcome related to the failure, Figure S4: Funnel plots of standard error with RR and pseudo 95% confidence limits that used to evaluate publication bias.
