# Supplementary material for: Tuberculosis Treatment Outcome and Predictors in Africa: A Systematic Review and Meta-Analysis
Source: Int J Environ Res Public Health. 2021 Oct 12;18(20):10678. doi: 10.3390/ijerph182010678 (PMC8536006; doi:10.3390/ijerph182010678)
Supplement: Supplementary file 1 [file ijerph-18-10678-s001.zip › ijerph-1355362-supplementary-FIGURE.pdf]

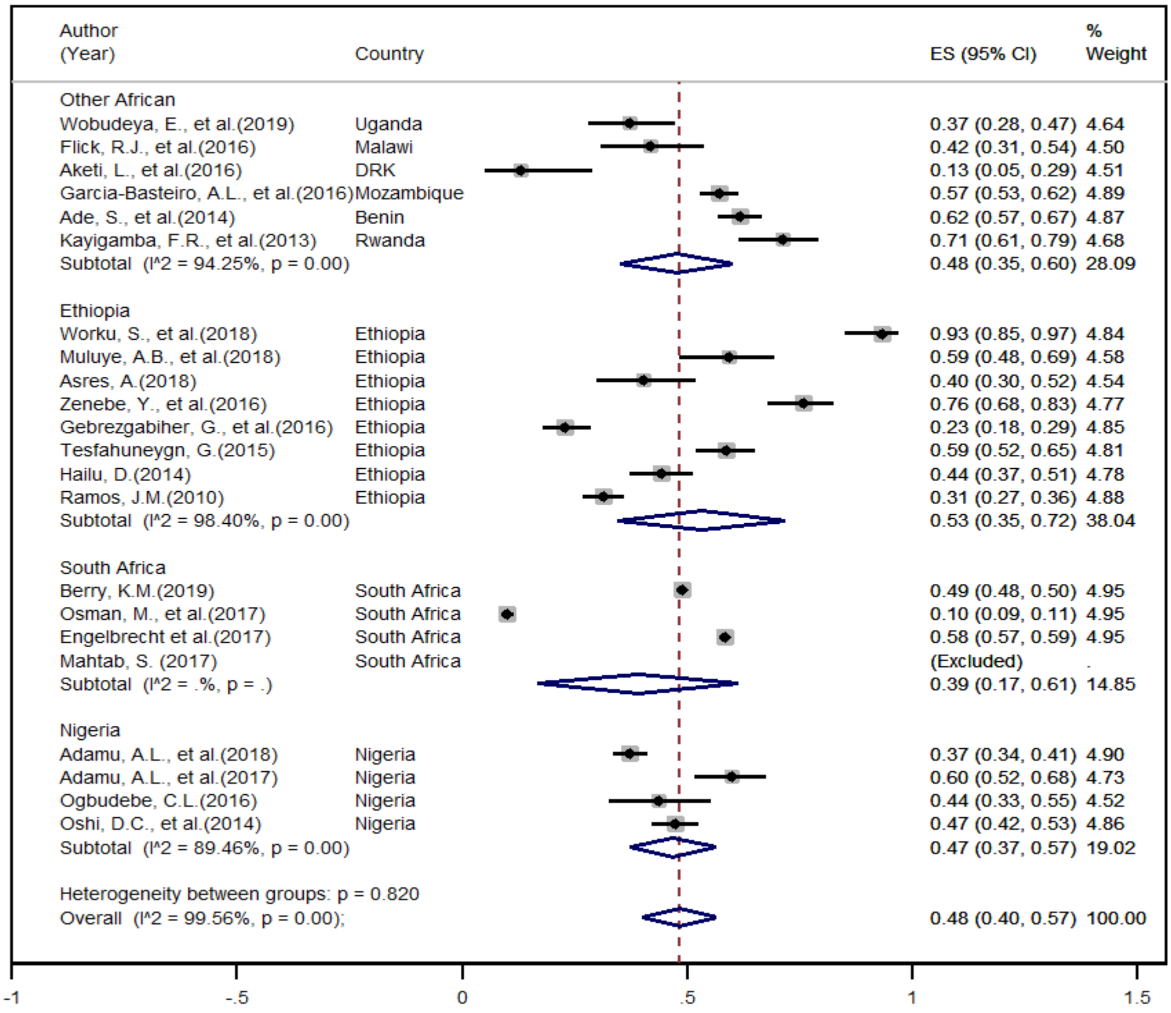


**Figure S1: Pooled and study-specific rate of unsuccessful treatment outcome related to death**

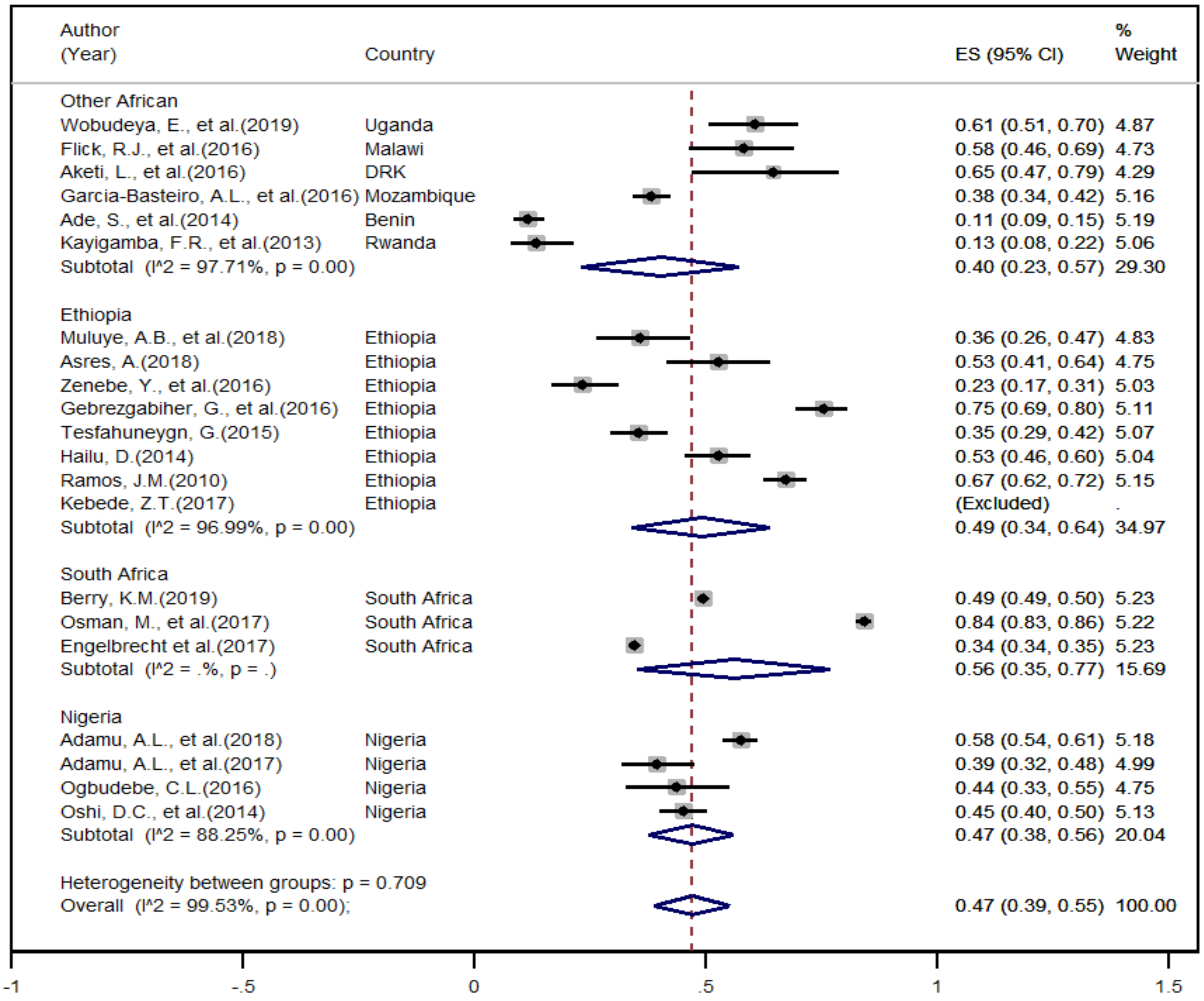


**Figure S2: Pooled and study-specific rate of unsuccessful treatment outcome related to the defaulter.**

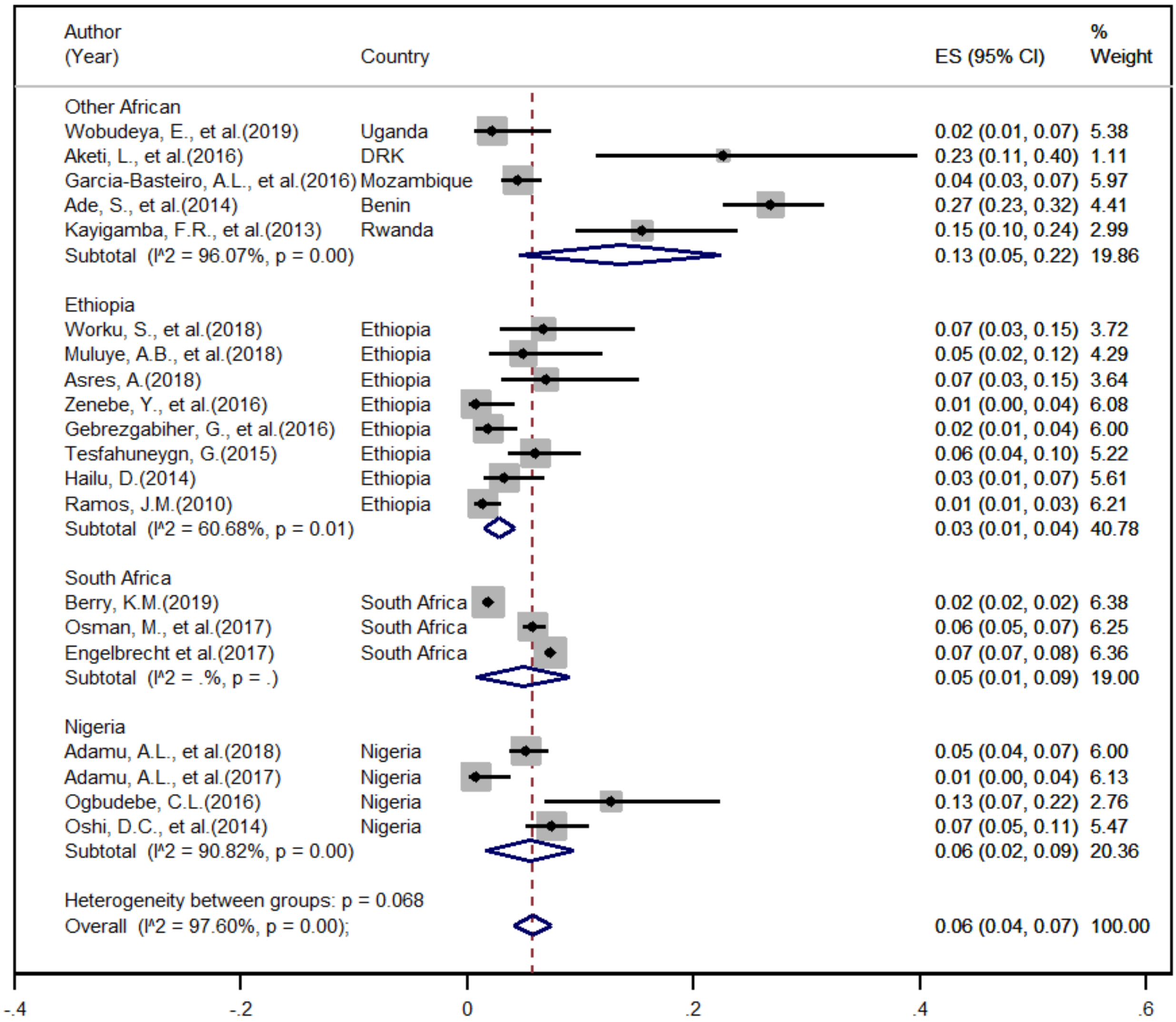


**Figure S3: Pooled and study-specific rate of unsuccessful treatment outcome related to the failure.**

Supplementary Figure 4:

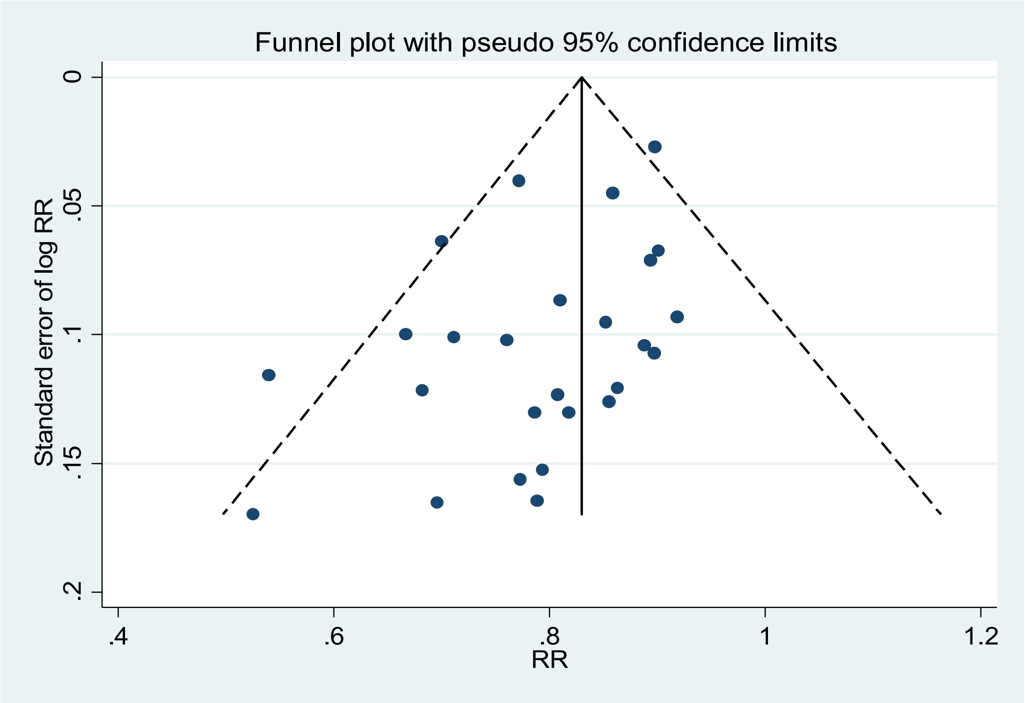


Figure S4: Funnel plots of standard error with RR and pseudo 95% confidence limits that used to evaluate publication bias
