# Supplementary material for: Tuberculosis Treatment Outcome and Predictors in Africa: A Systematic Review and Meta-Analysis
Source: Int J Environ Res Public Health. 2021 Oct 12;18(20):10678. doi: 10.3390/ijerph182010678 (PMC8536006; doi:10.3390/ijerph182010678)
Supplement: Supplementary file 1 [file ijerph-18-10678-s001.zip › Suplimentary files.pdf]

File Supplementary 1, Systematic Electronic search Term

((((((((((((((Determinants) OR Factors) OR "Risk factors"[MeSH Terms]) OR "Risk factors") OR Cause) OR Reason)) AND ((((((((Determinants) OR "Epidemiologic factors"[MeSH Terms]) OR "Epidemiologic factors") OR "Risk Factors"[MeSH Terms]) OR "Risk Factors") OR Factors) OR cause) OR reason)) AND ((((Lung[MeSH Terms]) OR Lung) OR Pulmonary) OR "Extra Pulmonary")) OR ((((Tuberculosis[MeSH Terms]) OR Tuberculosis) OR TB) OR Mycobacterium)) AND (((((Infection[MeSH Terms]) OR Infection) OR Disease[MeSH Terms]) OR Disease) OR Disorder)) AND ((((((((("Treatment outcome"[MeSH Terms]) OR "Treatment outcome") OR "Treatment adherence") OR Default) OR "Lost to follow up") OR "Treatment Failure") OR Refusal) OR "Transfer to care") OR Death)) AND (((Developing) OR "Resource limited") OR Poor)) AND (((Country) OR Nation) OR Region)))

Table S1. (Supporting information); Shows study level risk of bias measurement score using the Joanna Briggs Institute (JBI) critical appraisal assessment tool.

**Included studies**	**Reviewer #1**	**Total No Yes (Y)**	**Reviewer #2**	**Total No Yes (Y)**	**Average Yes (Y)**	**Percentage of Yes (Y)**	**Judgment**
Wobudeya, E., et al.(2019)	Y	7	Y	7	7	77.80%	Low
Mirutse, G., et al.(2019)	Y	7	Y	6	6.5	72.20%	Low
Berry, K.M.(2019)	Y	7	Y	7	7	77.80%	Low
Worku, S., et al.(2018)	Y	6	Y	7	6.5	72.20%	Low
Muluye, A.B., et al.(2018)	Y	7	Y	7	7	77.80%	Low
Asres, A.(2018)	Y	7	Y	6	6.5	72.20%	Low
Adamu, A.L., et al.(2018)	Y	6	Y	6	6	66.70%	Moderate
Pizzol, D., et al.(2018)	Y	6	Y	6	6	66.70%	Moderate
Osman, M., et al.(2017)	Y	8	Y	8	8	88.90%	Low
Engelbrecht et al.(2017)	Y	8	Y	8	8	88.90%	Low
Mahtab, S. (2017)	Y	8	Y	7	7.5	83.30%	Low
Kebede, Z.T.(2017)	Y	6	Y	6	6	66.70%	Moderate
Adamu, A.L., et al.(2017)	Y	6	Y	6	6	66.70%	Moderate
Flick, R.J., et al.(2016)	Y	6	Y	6	6	66.70%	Moderate
Aketi, L., et al.(2016)	Y	6	Y	6	6	66.70%	Moderate
Zenebe, Y., et al.(2016)	Y	6	Y	6	6	66.70%	Moderate
Tilahun, G.(2016)	Y	6	Y	6	6	66.70%	Moderate
Garcia-Basteiro, A.L., et al.(2016)	Y	8	Y	7	7.5	83.30%	Low
Ogbudebe, C.L.(2016)	Y	7	Y	7	7	77.80%	Low
Gebrezgabiher, G., et al.(2016)	Y	8	Y	8	8	88.90%	Low
Tesfahuneygn, G.(2015)	Y	7	Y	7	7	77.80%	Low
Hailu, D.(2014)	Y	8	Y	7	7.5	83.30%	Low
Oshi, D.C., et al.(2014)	Y	7	Y	7	7	77.80%	Low
Ade, S., et al.(2014)	Y	8	Y	7	7.5	83.30%	Low
Kayigamba, F.R., et al.(2013)	Y	6	Y	7	6.5	72.20%	Low
Ramos, J.M.(2010)	Y	7	Y	7	7	77.80%	Low
**Subtotal**							
**Y = Yes**			**75.43%**				
**N = No**			**24.62%**				

Note. Study level risk of bias is calculated from the domain of nine criteria.
